# Induction of Apoptosis in MCF-7 Cells via Oxidative Stress Generation, Mitochondria-Dependent and Caspase-Independent Pathway by Ethyl Acetate Extract of *Dillenia suffruticosa* and Its Chemical Profile

**DOI:** 10.1371/journal.pone.0127441

**Published:** 2015-06-05

**Authors:** Yin Sim Tor, Latifah Saiful Yazan, Jhi Biau Foo, Agustono Wibowo, Norsharina Ismail, Yoke Kqueen Cheah, Rasedee Abdullah, Maznah Ismail, Intan Safinar Ismail, Swee Keong Yeap

**Affiliations:** 1 Laboratory of Molecular Biomedicine, Institute of Bioscience, Universiti Putra Malaysia, 43400 UPM, Serdang, Selangor, Malaysia; 2 Department of Biomedical Science, Faculty of Medicine and Health Sciences, Universiti Putra Malaysia, 43400 UPM, Serdang, Selangor, Malaysia; 3 Department of Veterinary Pathology and Microbiology, Faculty of Veterinary Medicine, Universiti Putra Malaysia, 43400 UPM, Serdang, Selangor, Malaysia; 4 Department of Chemistry, Faculty of Science, Universiti Putra Malaysia, 43400 UPM, Serdang, Selangor, Malaysia; 5 Laboratory of Vaccines and Immunotherapeutics, Institute of Bioscience, Universiti Putra Malaysia, 43400 UPM, Serdang, Selangor, Malaysia; University of Windsor, CANADA

## Abstract

*Dillenia suffruticosa*, which is locally known as *Simpoh air*, has been traditionally used to treat cancerous growth. The ethyl acetate extract of *D*. *suffruticosa* (EADs) has been shown to induce apoptosis in MCF-7 breast cancer cells in our previous study. The present study aimed to elucidate the molecular mechanisms involved in EADs-induced apoptosis and to identify the major compounds in the extract. EADs was found to promote oxidative stress in MCF-7 cells that led to cell death because the pre-treatment with antioxidants α-tocopherol and ascorbic acid significantly reduced the cytotoxicity of the extract (P<0.05). DCFH-DA assay revealed that treatment with EADs attenuated the generation of intracellular ROS. Apoptosis induced by EADs was not inhibited by the use of caspase-inhibitor Z-VAD-FMK, suggesting that the cell death is caspase-independent. The use of JC-1 dye reflected that EADs caused disruption in the mitochondrial membrane potential. The related molecular pathways involved in EADs-induced apoptosis were determined by GeXP multiplex system and Western blot analysis. EADs is postulated to induce cell cycle arrest that is p53- and p21-dependent based on the upregulated expression of p53 and p21 (P<0.05). The expression of Bax was upregulated with downregulation of Bcl-2 following treatment with EADs. The elevated Bax/Bcl-2 ratio and the depolarization of mitochondrial membrane potential suggest that EADs-induced apoptosis is mitochondria-dependent. The expression of oxidative stress-related AKT, p-AKT, ERK, and p-ERK was downregulated with upregulation of JNK and p-JNK. The data indicate that induction of oxidative-stress related apoptosis by EADs was mediated by inhibition of AKT and ERK, and activation of JNK. The isolation of compounds in EADs was carried out using column chromatography and elucidated using the nuclear resonance magnetic analysis producing a total of six compounds including 3-epimaslinic acid, kaempferol, kaempferide, protocatechuic acid, gallic acid and β-sitosterol-3-O-β-D-glucopyranoside. The cytotoxicity of the isolated compounds was determined using MTT assay. Gallic acid was found to be most cytotoxic against MCF-7 cell line compared to others, with IC_50_ of 36 ± 1.7 μg/mL (P<0.05). In summary, EADs generated oxidative stress, induced cell cycle arrest and apoptosis in MCF-7 cells by regulating numerous genes and proteins that are involved in the apoptotic signal transduction pathway. Therefore, EADs has the potential to be developed as an anti-cancer agent against breast cancer.

## Introduction

There is a shift of attention in chemotherapy from the use of a single drug to multi-drugs for the management of various kinds of cancer, which aims to regulate diverse signaling processes responsible for the survival of tumor via suppression or activation of multiple targets simultaneously [1]. The same concept applies to phytotherapy, where plant extract contains a variety of bioactive compounds that may exert synergistic effect for cancer therapy [2, 3]. Since cancer is a complex disease characterized by alteration in dynamic and complicated signaling pathways that modulate cell growth, survival, differentiation and invasion, the richness of constituents in plant extract may act or target different receptors of the signaling pathways, thus improving the therapeutic effect compared to a single compound treatment [4, 5].

Failure to induce apoptosis is a crucial factor that leads to the formation of cancer [6]. Hence, the capability of an anticancer candidate to control cell death and survival through induction of apoptosis is of great advantage for the management of the disease [7]. Apoptosis is triggered by interconnected signaling pathways and modulated by diversified target molecules. Apoptosis may occur through either mitochondria-dependent or mitochondria-independent pathway [8].

Mitochondria-mediated or intrinsic pathway is controlled by the family of Bcl-2 proteins. The roles of pro-apoptotic and anti-apoptotic Bcl-2 family of proteins are significant in maintaining the permeability of mitochondrial membrane, thus governing the activation or abortion of apoptosis [9]. Caspases are often considered as the executioners of apoptosis. Nevertheless, recent findings suggest that apoptosis can also occur without the presence of caspases, but via other proteases as cell death executioners [10]. Another important regulator of apoptosis is the tumor suppressor, p53, which can also incite DNA repair, cell cycle checkpoints and cellular senescence [11]. p53 can also control the transcription of the members of Bcl-2 family especially Bcl-2 and Bax. Besides, p53 is able to activate the transcription of p21, a cyclin-dependent kinase inhibitor, during DNA damage that can influence the cell cycle progression by interacting with different transcription factors and lead to apoptosis [12, 13].

Another signaling pathway that plays an important role in apoptosis is Jun N-terminal kinase (JNK) pathway. It can promote apoptosis either by the regulation of pro-apoptotic genes through distinct transcription factor transactivation in nuclear signaling or by the manipulation of pro- and anti-apoptotic proteins in mitochondria [14]. In contrast to JNK pathway, extracellular signal-regulated kinase (ERK) pathway is associated with proliferation, survival and differentiation of cells [15]. Meanwhile, PI3-K/AKT pathway regulates cell survival, metabolism, cell growth and angiogenesis [16]. Inhibition of AKT and ERK pathway will elicit apoptosis in cancerous cells [17].

Our previous study demonstrated that EADs inhibited the growth of breast cancer cells through induction of apoptosis by regulating numerous genes such as *SOD1*, *SOD2*, *catalase*, *Akt1*, *NF-κB*, *p53*, and *p38 MAPK* [18, 19]. This study further investigated the role of oxidative stress and involvement of numerous genes and proteins in the signal transduction pathways of EADs-induced apoptosis. In addition, the isolation and identification of compounds from EADs were reported for the first time in this study. Collectively, it is believed that better understanding of the mechanisms underlying the anticancer activities of EADs and the cytotoxic effect of its constituents would facilitate the development of promising cancer therapeutics using herbal medicine.

## Materials and Methods

### Plant Material

*D*. *suffruticosa* plant with voucher specimen number SK1937/11 was deposited in the herbarium of Institute of Bioscience, Universiti Putra Malaysia. The fine root powder of *D*. *suffruticosa* was supplied by Primer Herber Sdn. Bhd. (Terengganu, Malaysia).

### Chemicals

Hexane, dichloromethane, ethyl acetate, methanol, acetone and chloroform were obtained from FS Chemicals (Francfort, Germany). Silica gel 60 (0.063–0.200 mm), thin layer chromatography, preparative chromatography, NMR grade chloroform, methanol and DMSO were obtained from Merck (Darmstadt, Germany). RPMI 1640 without phenol red and Dulbecco’s Modified Eagle’s Medium (DMEM) were purchased from Nacalai Tesque (Kyoto, Japan). Fetal bovine serum and horse serum were supplied by JR Scientific Inc. (California, USA). Trypsin, streptomycin and penicillin were obtained from PAA Laboratories GmBH (Pasching, Austria). 3-(4,5-dimethylthiazol-2-yl)-2,5 diphenyltetrazolium bromide (MTT), cholera toxin, human insulin, epidermal growth factor and hydrocortisone were purchased from Sigma (St. Loius, USA). Tissue culture flasks, 6-well plates and 96-well plates were obtained from TPP Techno Plastic Products (Trasadingan, Switzerland). JC-1 Mitochondrial Membrane Potential Assay Kit was procured from Cayman Chemicals (Ann Arbor, Michigan, USA). Real Genomics Total RNA Extraction Kit (RBC Biosciences, Taiwan) and GenomeLab GeXP Start Kit (Beckman Coulter, USA) were also purchased. Phenylmethanesulfonyl fluoride (PMSF) and protease inhibitor cocktails were purchased from Calbiochem (California, USA). Phosphatase inhibitor cocktails, bovine serum albumin (BSA) and Chemi-Lumi One L were purchased from Nacalai Tesque (Kyoto, Japan). Sodium dodecyl sulphate (SDS), Triton-X 100, Tris-base, glycine, acrylamide, bisacrylamide, ammonium persulfate (APS), tetramethylethylenediamine (TEMED), 10% Tween-20, Bradford reagent, 2-mercaptoethanol, extra thick blotting paper and pre-stained protein marker were purchased from Bio-Rad (California, USA). Immobilon-FL polyvinylidene fluoride (PVDF) membrane (pore size 0.45 μm) was purchased from Millipore (Massachusetts, USA). All primary antibodies and secondary antibodies were purchased from ABCAM (Massachusetts, USA).

### Extraction of EADs

Sequential solvent extraction was performed by using various solvents of increasing polarity (hexane, dichloromethane and ethyl acetate). Briefly, 1 kg of the root powder of *D*. *suffruticosa* was macerated in hexane at a ratio of 1:5 (w/v) with occasional shaking using a rotary shaker, for 24 hours, at room temperature (25 ± 2°C). Next, the mixture solvent was filtered using Whatman No. 1 filter paper and the residue was re-extracted for three times. The filtrates were collected and pooled together. The residue of filtrates was dried in an oven at 40°C for 24 hours. Subsequently, the residue was reused for successive extraction using dichloromethane (DCM) (1:5, w/v) followed by ethyl acetate (EtOAc) (1:5, w/v) using the same methods. Lastly, ethyl acetate filtrates were evaporated using a vacuum rotary evaporator (Rotavapor R210, Buchi, Switzerland) to remove ethyl acetate and the yield was dried in an oven (40°C) overnight. EtOAc was completely removed when constant weight was obtained. The final yield was weighed and kept at -20°C until further use. [18]

### Cell culture

The human adenocarcinoma breast cancer cell lines, hormone-dependent MCF-7 (Catalog Number: HTB-22) and hormone-independent MDA-MB-231 (Catalog Number: HTB-26D), and human epithelial mammary gland cell line, MCF-10A (Catalog Number: CRL-10317) were obtained from the American Type and Culture Collection (Rockville, USA). MCF-7 and MDA-MB-231 cells were cultured in RPMI 1640 supplemented with 10% fetal bovine serum and 1% penicillin and streptomycin. MCF-10A cells were cultured in DMEM supplemented with 5% horse serum, 1 ng/mL of cholera toxin, 10 μg/ mL of human insulin, 10 ng/mL of epidermal growth factor, 0.5 μg/mL of hydrocortisone and 1% penicillin/streptomycin. The cells were maintained in a humidified incubator at 37°C in atmosphere of 5% CO_2_.

### Evaluation of the induction of oxidative stress by EADs in MCF-7 cells

MCF-7 cells were seeded at a density of 5,000 cells per well of a 96-well plate and incubated overnight for cell attachment. Cells were then pre-treated with 50 μM α-tocopherol or 0.1 mM ascorbic acid for 6 hours before treatment with EADs (3.13–100 μg/mL). Untreated control cells were included. After 24 and 48 hours, 20 μL of 5 mg/mL of MTT was added into each well, and the plate was incubated for 3 hours. Next, media in each well was discarded and 100 μL of DMSO was added to solubilize the purple blue formazan [20]. The absorbance was measured with ELx800 Absorbance Microplate Reader (Biotek Instruments Inc., Vermont, USA) at wavelength of 570 nm, and 630 nm as reference wavelength. A graph of percentage of cell viability versus concentration of EADs was plotted, and the IC_50_ (concentration of EADs that inhibits 50% of cell growth compared to the control) was determined.

### Determination of the involvement of reactive oxygen species in EADs-induced oxidative stress

The level of intracellular reactive oxygen species (ROS) was determined using dichlorodihydrofluorescein diacetate assay (DCFH-DA, Sigma-Aldrich, St. Louis, MO, USA). Briefly, MCF-7 cells were seeded at a density of 5,000 cells per well of a 96 well plate and incubated overnight for cell attachment. Next, the cells were washed with 1X PBS and incubated with 10 μM of DCFH-DA for one hour. Subsequently, the cells were rinsed with PBS and treated with either 25 or 50 μg/mL of EADs, or 50 μM of H_2_O_2_ solution (positive control) for 3 hours. DCF fluorescence intensity was measured using Synergy-4 microplate reader (Biotek Instruments Inc., Vermont, USA) at excitation wavelength of 485 nm and emission wavelength of 535 nm.

### Determination of the involvement of caspase in EADs-induced apoptosis

MCF-7 cells were seeded at a density of 200,000 cells per well of a 6-well plate and incubated overnight for cell attachment. Next, MCF-7 cells were pre-treated with 50 μM of caspase inhibitor Z-VAD-FMK (R&D Systems Inc., Minneapolis, USA) for 2 hours at 37°C and 5% of CO_2_. Subsequently, the cells were treated with EADs at 25 and 50 μg/mL. Control untreated cells were included. After incubation for 48 hours, the floating cells in culture media were collected and the cells attached to the substratum were trypsinized. The cells were centrifuged at 100 x *g* and the supernatant was discarded. The cells were washed twice with PBS. Subsequently, 185 μL of 1X binding buffer, 5 μL of Annexin-V FITC and 10 μL of propidium iodide (PI) were added into the pellet and incubated at room temperature (25°C) for 10 minutes in the dark. Next, 300 μL of 1X binding buffer was added prior to measurement using FACS calibur flowcytometer and Cell Quest Pro software (BD Biosciences, USA). The fluorescence colour was detected at excitation wavelength of 530 nm and emission wavelength of 585 nm. A total of 10,000 cells were acquired. The data were analyzed using FlowJo 7.6 software and displayed in dot plot of Annexin V/FITC (Y-axis) against PI (X-axis).

### Determination of the effect of EADs on mitochondrial membrane potential

The Cayman JC-1 Assay Kit (Cayman Chemicals Company, Ann Arbor, Michigan, USA) was used to measure the alteration of the mitochondria membrane potential (ΔΨm). JC-1 forms aggregates, which emit red fluorescence in the mitochondria of healthy cells. However, it remains as monomers that emit green fluorescence during the loss of ΔΨm. Briefly, MCF-7 cells were seeded at a density of 500,000 cells in a 25 cm^2^ culture flask and were incubated overnight for cell attachment. The cells were then treated with 25 and 50 μg/mL of EADs. The untreated control cells were included. After 24 and 48 hours, 100 μL of JC-1 Staining Solution (1:10 dilution in culture medium) was added into each 1 mL of culture medium and incubated for 15 minutes at 37°C in a CO_2_ incubator. Next, the cells were trypsinized and collected by centrifugation at 100 x *g* for 5 minutes. The cells were washed twice with 1 mL of Assay Buffer. Subsequently, 500 μL of Assay Buffer was added to the cell pellet in each tube and resuspended well. The samples were analyzed immediately using FACS calibur flow cytometer and Cell Quest Pro software (BD Biosciences, USA). A total of 10,000 cells were acquired. The data were analyzed using FlowJo 7.6 software and displayed in dot plot of JC-1 red fluorescence (Y-axis) against JC-1 green fluorescence (X-axis).

### Determination of the effects of EADs on the expression of apoptotic pathway–related genes

#### RNA isolation

MCF-7 cells were seeded at a density of 200,000 cells per well of a 6-well plate. After incubated overnight for cell attachment, the cells were treated with 25 and 50 μg/mL of EADs for 24 hours. Untreated control cells were included. After 24 hours, the cells were trypsinized and collected by centrifugation at 100 x *g* for 5 minutes. RNA extraction was carried out following the protocol of the Real Genomics Total RNA Extraction Kit (RBC Biosciences, Taiwan). Briefly, 400 μL of RB buffer, 100 μL of lysis buffer and 4 μL of β-mercaptoethanol were added into the pellet to lyse the cells. After 5 minutes, 400 μL of 70% ethanol was added into the cell lysate and mixed vigorously by using a pipette. RNA binding was performed by transferring the mixture to RT column and centrifuged at 1,000 x *g* for 2 minutes. Next, the RT column was washed once with W1 Buffer and twice with Wash Buffer. Next, 50 μL of RNAse-free water was added to the column matrix and centrifuged at 1,000 x *g* for 1 minute to obtain purified RNA sample. The concentration and quality of RNA were quantified using a nanophotometer (Implen, Baxter Avenue, Britain).

#### Reverse transcription and polymerase chain reaction

Reverse transcription reaction mixture was prepared according to the GenomeLab GeXP Start Kit (Beckman Coulter, USA). Briefly, 11 μL of RNA-free water was mixed with 2 μL of customized reverse primer of the desired gene, 1 μL of reverse transcriptase, 4 μL of reverse transcription buffer and 1 μL of 50 ng/μL of RNA sample isolated previously. The reverse transcription reaction was performed as follow: primer annealing at 48°C for 1 minute, reverse transcription at 42°C for 60 minutes and denaturation at 95°C for 5 minutes. Subsequently, the cDNA product was amplified by polymerase chain reaction (PCR) reaction. The PCR reaction mixture, which consists of 4 μL of 5X PCR buffer, 4 μL of MgCl_2_, 2 μL of customized forward primer mixture (Table 1), 0.7 μL of *Taq* polymerase and 9.3 μL of cDNA was prepared. Next, PCR was performed as follow: denaturation of DNA at 95°C for 10 minutes, DNA annealing at 94°C and 55°C, both for 30 seconds, and elongation of DNA at 70°C for 1 minute. The second and third step were repeated for a total of 35 thermal cycles. Lastly, the PCR tube with sample was hold at 4°C.

**Table 1:** List of genes with their respective primers for GeXP multiplex analysis.

Gene	Forward primer sequence	Reverse primer sequence
*Bcl-2*	5’-ACCACTAATTGCCAAGCACC-3’	3’-ATTTTCCATCCGTCTGCTCTT-5’
*Bax*	5’-CCCTTTTGCTTCAGGGTTTC-3’	3’-ACAAAGTAGAAAAGGGCGACAA-5’
*p21*	5’-TTAGCAGCGGAACAAGGAGT-3’	3’-AAGCCGAGAGAAAACAGTCCA-5’
*PARP1*	5’-TATCGAGTCGAGTACGCCAA-3’	3’-AAACTACCTTTTCAGGGTGTG-5’
*Beta actin*	5’-GATCATTGCTCCTCCTGAGC-3’	3’-AAAAGCCATGCCAATCTCATC-5’
Kan(r)^b^	5’-ATCATCAGCATTGCATTCGAT TCCTGTTTG-3’	3’-AATTCCGACTCGTCCAACATC-5’

Forward universal primer sequence (5’-AGGTGACACTATAGAATA-3’); Reverse universal primer sequence (3’-GTACGACTCACTATAGGG-5’).

#### GeXP multiplex analysis

The GenomeLab GeXP genetic analysis system (Beckman Coulter, USA) was used to investigate the multiplex of gene expression in EADs-treated MCF-7 cells. The primers of all genes were supplied by First Base Ltd. (Selangor, Malaysia). Briefly, 1 μL of PCR product was mixed with 38.5 μL of sample loading solution and 0.5 μL of DNA Size Standard 400. The mixture was added into a 96-well sample plate and run using GeXP Genetic Analysis System (Beckman Coulter, USA). The amplified fragments were separated according to their respective size by capillary gel electrophoresis in the GeXP system. Results were analyzed using the Fragment Analysis module of the GeXP system software and eXpress Profiller software. The expression of the genes was normalized against beta actin.

### Determination of the effects of EADs on the expression of apoptotic pathway-related proteins

MCF-7 cells were seeded at a density of 800,000 cells in a 75 cm^2^ culture flask and were incubated overnight for cell attachment. The cells were then treated with 25 and 50 μg/mL of EADs. The untreated control cells were included. After 24 and 48 hours, the cells were trypsinized, washed with PBS and collected by centrifugation at 100 x *g* for 5 minutes. The cells were lysed with 200 μL of ice cold RIPA lysis buffer (50 mM Tris-HCL pH 7.4, 150 mM NaCl, 0.1% SDS (w/v), 0.5% sodium deoxycholate (w/v), 1% Triton X-100 (v/v), 1 mM PMSF, 10 μL/mL phosphatase inhibitor cocktails and 10 μL/mL of protease inhibitor cocktail). Next, cell lysate was collected and centrifuged at 1,000 x *g* for 10 minutes at 4°C. The supernatant was collected and stored at -80°C until further use. The protein concentration of the samples was determined by using Bradford assay (BioRad, CA, USA) [21]. Equal amount (10–20 μg) of protein sample was mixed with 4X Laemmlli buffer (10% β-mercaptoethanol) at the ratio of 3:1 and heated at 95°C for 5 minutes. The sample was loaded into each well of the SDS-PAGE gel. Electrophoresis was run at 50V in 4% stacking gel, and at 100V in 12% of resolving gel.

Semi dry-transfer method was employed to transfer the proteins onto PVDF membrane (Milipore, Bedford, MA, USA) under constant voltage (12V). The membrane was blocked with 3% bovine serum albumin (Nacalai Tesque, Japan) in Tris buffer saline containing 0.1% of Tween-20 (TBS-T). After blocking for 1 hour at room temperature, target proteins were probed overnight under constant shaking at 4°C with their respective primary antibodies. Monoclonal mouse anti-p53 (DO-1) was obtained from Santa Cruz (Dallas, Texas, USA). Polyclonal rabbit anti-p-AKT-1 was procured from Cell Signalling Technology, Inc. (Danvers, MA, USA). Other antibodies such as polyclonal rabbit anti-p21 (ab7960), monoclonal mouse anti-Bax (ab5714), polyclonal rabbit anti-Bcl-2 (ab7973), polyclonal rabbit anti-caspase 8 (ab25901), monoclonal rabbit anti-AKT-1 (ab32505), polyclonal rabbit anti-JNK-1 (ab10664), polyclonal rabbit anti-p-JNK-1 (ab47337), monoclonal rabbit anti-ERK-1 (ab32537), polyclonal rabbit anti-p-ERK-1 (ab47310), polyclonal rabbit anti-PARP (ab137653) and monoclonal mouse anti-beta actin (ab8226) were obtained from ABCAM (Cambridge, MA, USA). Next, the membrane was washed with TBS-T for 5 times and incubated with peroxidase-conjugated secondary goat anti-rabbit (ab6721) or goat anti-mouse (ab97240) antibodies from ABCAM (Cambridge, MA, USA) for 1 hour at room temperature. The chemiluminiscent signals were detected using Chemi Lumi-One L Detection Reagent (Nacalai Tesque, Japan) and ChemiDoc MP System (Bio-Rad, Hercules, CA, USA).

### Isolation of compounds of EADs

**Fig 1:**
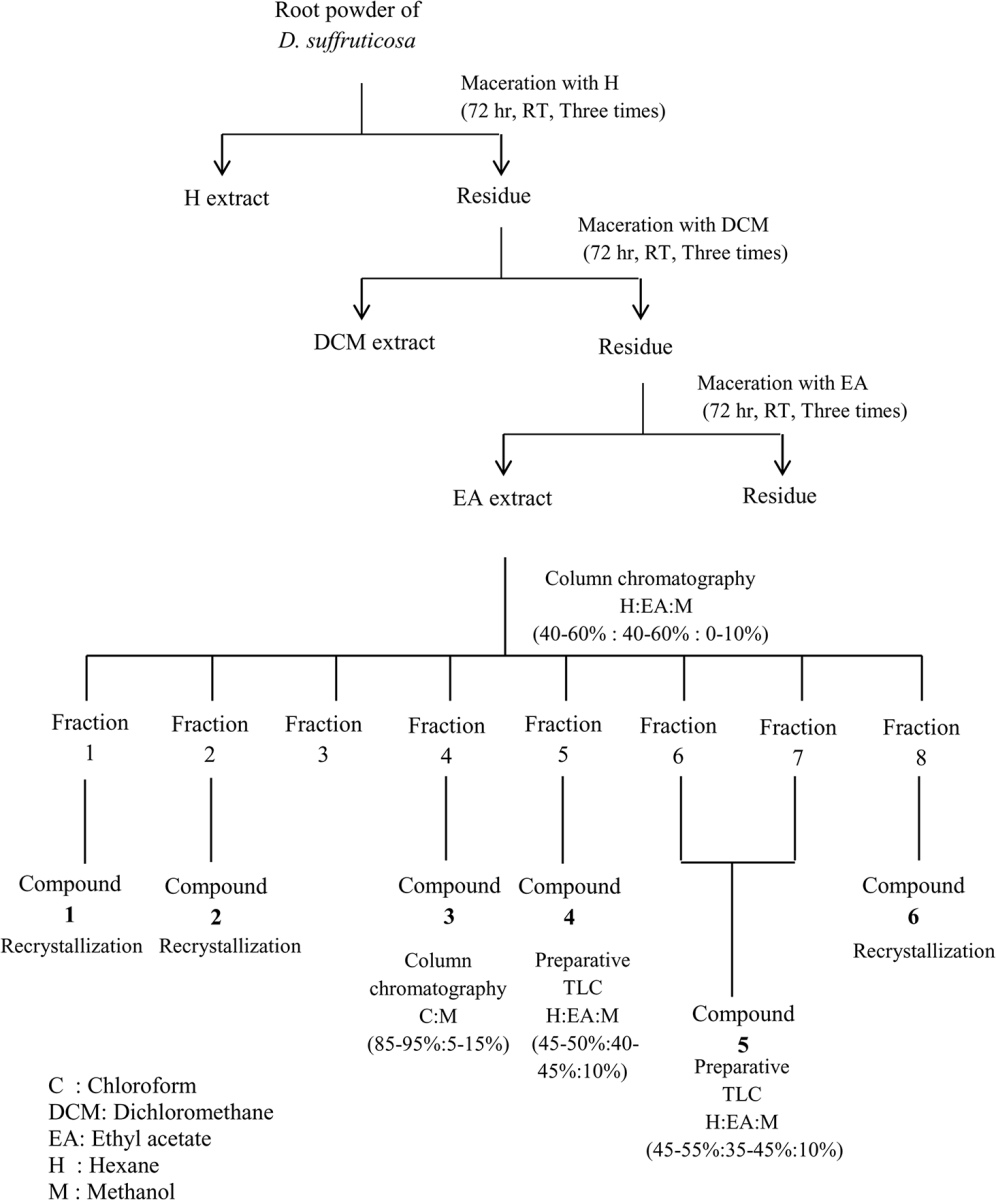
Extraction and isolation of ethyl acetate extract of *Dillenia suffruticosa*. Sequential solvent extraction using solvents with increasing polarity (hexane< dichloromethane<ethyl acetate) was carried out to obtain ethyl acetate fraction of *D*. *suffruticosa*. The extract obtained was subjected to isolation using column chromatography and thin layer chromatography by using different solvent systems.

Extraction of EADs was performed using the methods described above. Briefly, 4 kg of dried root powder of *D*. *suffruticosa* yielded 20 g of brown residue. The dried ethyl acetate extract (20 g) was dissolved in ± 200 mL of methanol. Diethyl ether was later added to a volume of ± 2 L to remove tannin constituents. After decantation, both methanol-diethyl ether soluble fraction and insoluble fraction were collected. Tannin forms precipitate in insoluble fraction, while the soluble fraction was evaporated for further use. As depicted in Fig 1, the soluble fraction (13 g) was subjected to liquid chromatography (silica gel, 200 g; eluted with mixtures of n-hexane/ethyl acetate 10% to 60% and n-hexane/ethyl acetate/methanol 10% to 20%) to give eight major fractions (F_1_–F_8_) (as shown in Fig 1). Fraction F_4_ was refractionated using column chromatography (eluent, chloroform:methanol 5 to 15%) and gave rise to ten fractions (F_4.1—_F_4.10_). Purification of fraction F_1_ and F_2_ yielded compound **1** (0.21%, 42 mg) and compound **2** (0.05%, 10 mg), respectively. Purification of fraction F_4.6_ yielded compound **3** (0.14%, 27 mg). Further purification of F_5_ using preparative thin layer chromatography (TLC mm; eluent, n-hexane:ethyl acetate:methanol = 5:4:1) gave rise to three fractions (F_5.1—_F_5.3_). Purification of F_5.3_ yielded compound **4** (0.57%, 113 mg). F_6_ and F_7_ were subjected to purification using preparative thin layer chromatography, eluted with n-hexane:ethyl acetate:methanol, (4.5:4.5:1) and (5:4:1), respectively. Five fractions from preparative thin layer chromatography were obtained separately, F_6.1_ and F_7.1_ yielded compound **5** (11.2%, 2.24 g). The purification of fraction F_8_ yielded compound **6** (0.96%, 192 mg). Detection was carried out by using UV torch and spraying of cerium (IV) sulphate, followed by heating at 105°C for 1–2 minutes on TLC (Merck, Kieselgel 60 F254 0.25 mm). ^1^H and ^13^C NMR spectra were measured using JEOL ECX 500 MHz Nuclear Magnetic Resonance Spectrophotometer (Peabody, MA, USA) carried out at 500 and 125 Mhz, respectively. Samples were dissolved either in CDCL_3_, CD_3_OD or DMSO-d_6_.

### Determination of cytotoxicity of the isolated compounds

MCF-7 and MDA-MB-231 cells were seeded at the density of 5,000 cells per well of a 96-well plate and incubated overnight for cell attachment. Cells were then treated with the isolated compounds (3.13–100 μg/mL). Untreated control cells were included. After treatment for 72 hours, 20 μL of 5 mg/mL of MTT was added into each well and the plate was incubated for 3 hours. Next, media in each well was discarded and 100 μL of DMSO was added to solubilize the purple blue formazan. The absorbance was measured with ELx800 Absorbance Microplate Reader (Biotek Instruments Inc., Vermont, USA) at wavelength of 570 nm, and 630 nm as reference wavelength [20]. The IC_50_ (concentration of isolated compounds that inhibits 50% of cell growth compared to the control) was determined and tabulated in a table that represents the IC_50_ ± SD of isolated compounds.

### Statistical Analysis

Data are represented as mean± SD of at least three independent experiments. Data were analyzed using IBM SPSS version 20. One way ANOVA and Tukey’s post hoc test were used for pairwise comparisons. P value less than 0.05 was considered statistically significant.

## Results

### EADs induced oxidative stress in MCF-7 cells

**Fig 2:**
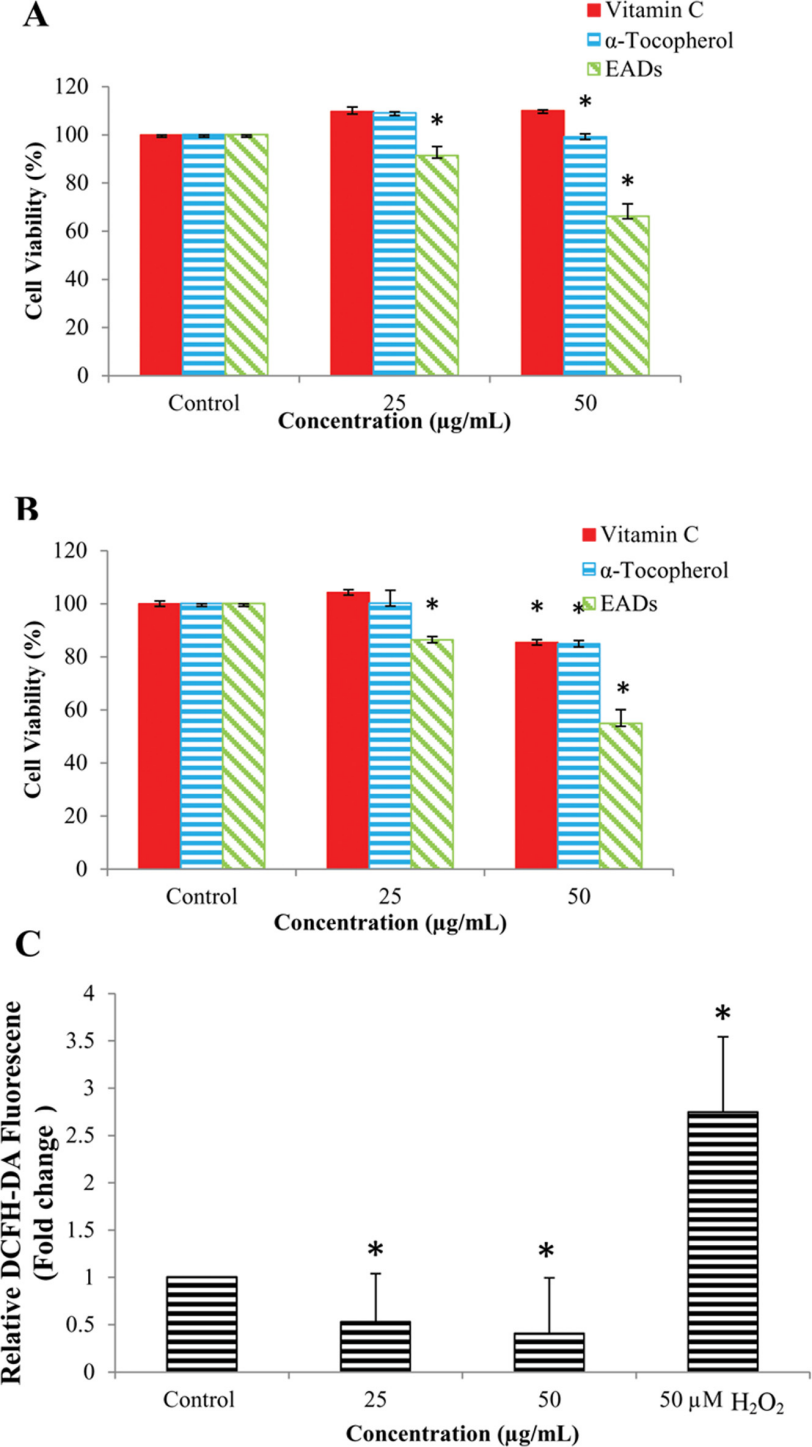
Involvement of oxidative stress in EADs-induced apoptosis in MCF-7 cells. (A) and (B) represent the percentage of viability of MCF-7 cells pre-treated with vitamin C, α-tocopherol or EADs alone, at 24 and 48 hours, respectively. (C) Level of ROS in MCF-7 cells as determined using DCFH-DA assay. Data showed that pre-treatment of MCF-7 cells with α-tocopherol and ascorbic acid significantly reduced the cytotoxicity of EADs (P<0.05). On the other hand, EADs attenuated the intracellular ROS in MCF-7 cells in a concentration-dependent manner (P<0.05). The data are presented as mean±standard deviation of three replicates from at least three independent tests. An asterisk * indicates statistically significantly different from the untreated control (P<0.05).

The involvement of oxidative stress in EADs-induced apoptosis was confirmed by pre-treatment of the cells with antioxidants prior to EADs treatment. As depicted in Fig 2A and 2B, 6 hours pre-treatment with α-tocopherol and ascorbic acid significantly increased the viability of MCF-7 cells treated with EADS in a time and dose-dependent manner to 110% and 99%, respectively, compared to the cells treated with 50 μg/mL of EADs alone (66%) at 24 hours (P<0.05). Similar trend was observed at 48 hours.

### EADs attenuated ROS formation in MCF-7 cells

Fluorescent probe DCFH-DA was used to measure the formation of intracellular ROS in EADs-induced apoptosis. Exposure of MCF-7 cells to 50 μM of hydrogen peroxide significantly increased the formation of ROS by 2.73 fold compared to the untreated control (P<0.05) (Fig 2C). Treatment of MCF-7 cells at 25 and 50 μg/mL of EADs significantly attenuated the formation of intracellular ROS by 48% and 60%, respectively (P<0.05).

### EADs induced caspase-independent apoptosis in MCF-7 cells

**Fig 3:**
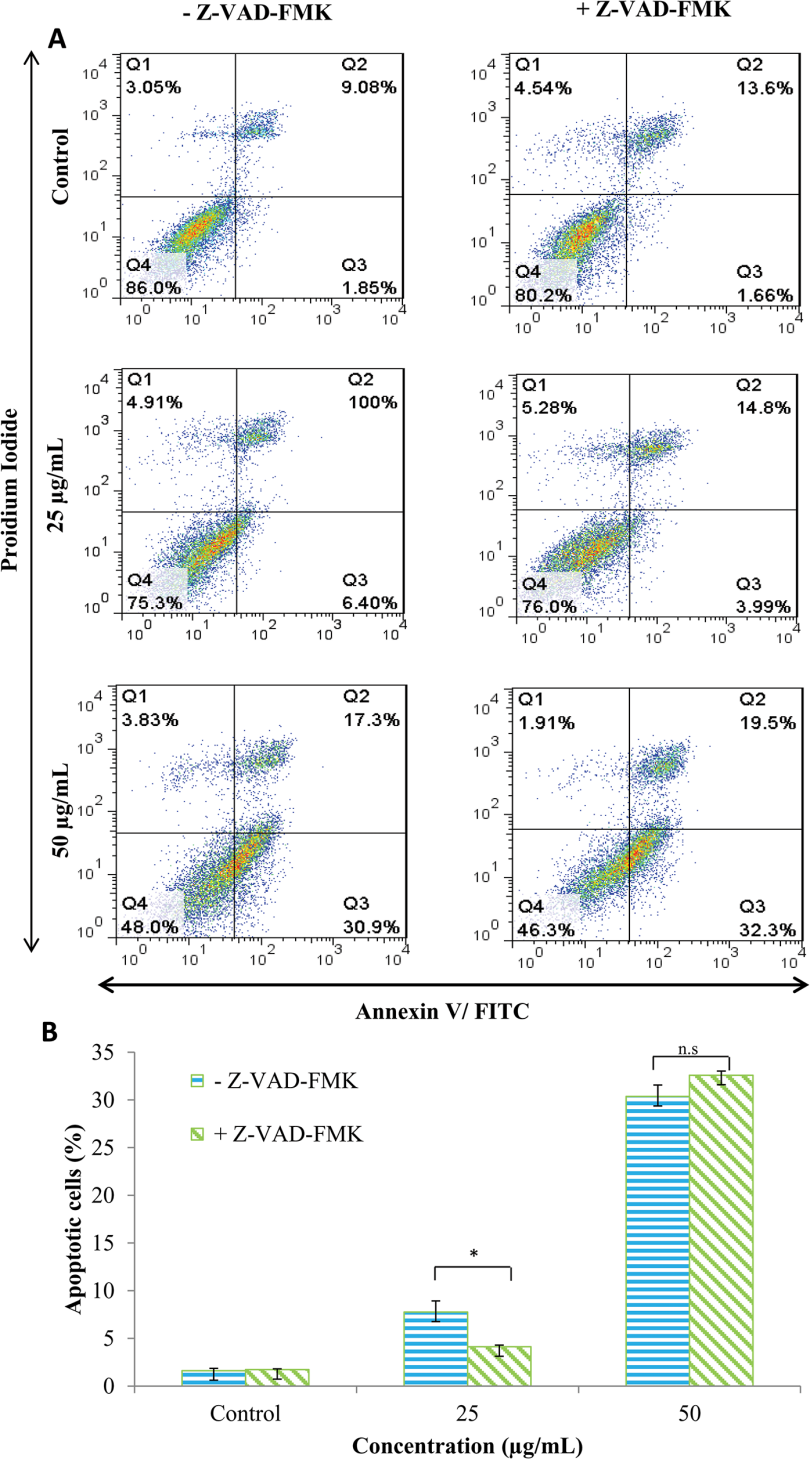
Involvement of caspase in EADs-induced apoptosis in MCF-7 cells. General inhibitor Z-VAD-FMK did not inhibit the induction of apoptosis by EADs suggesting it is caspase-independent. (A) represents mean percentage of three independent experiments±SD. (B) Comparison of the percentage of apoptotic cells between caspase inhibitor negative group and caspase inhibitor positive group at different concentrations. The data are presented as mean±standard deviation of three replicates from three independent tests. An asterisk * indicates statistically significantly different from the untreated control (P<0.05).

To further assess the involvement of caspases in EADs-induced apoptosis, general inhibitor, Z-VAD-FMK, was employed to probe whether it could protect MCF-7 cells from undergoing apoptosis. As shown in Fig 3, at 25 μg/mL of EADs, the percentage of early apoptotic cells in Z-VAD-FMK pre-treated group decreased compared to the Z-VAD-FMK untreated group (P<0.05). However, at 50 μg/mL of EADs, the percentage of early apoptotic cells in both Z-VAD-FMK pre-treated and untreated groups was not significantly different (P>0.05).

### EADs induced depolarization of mitochondrial membrane potential

**Fig 4:**
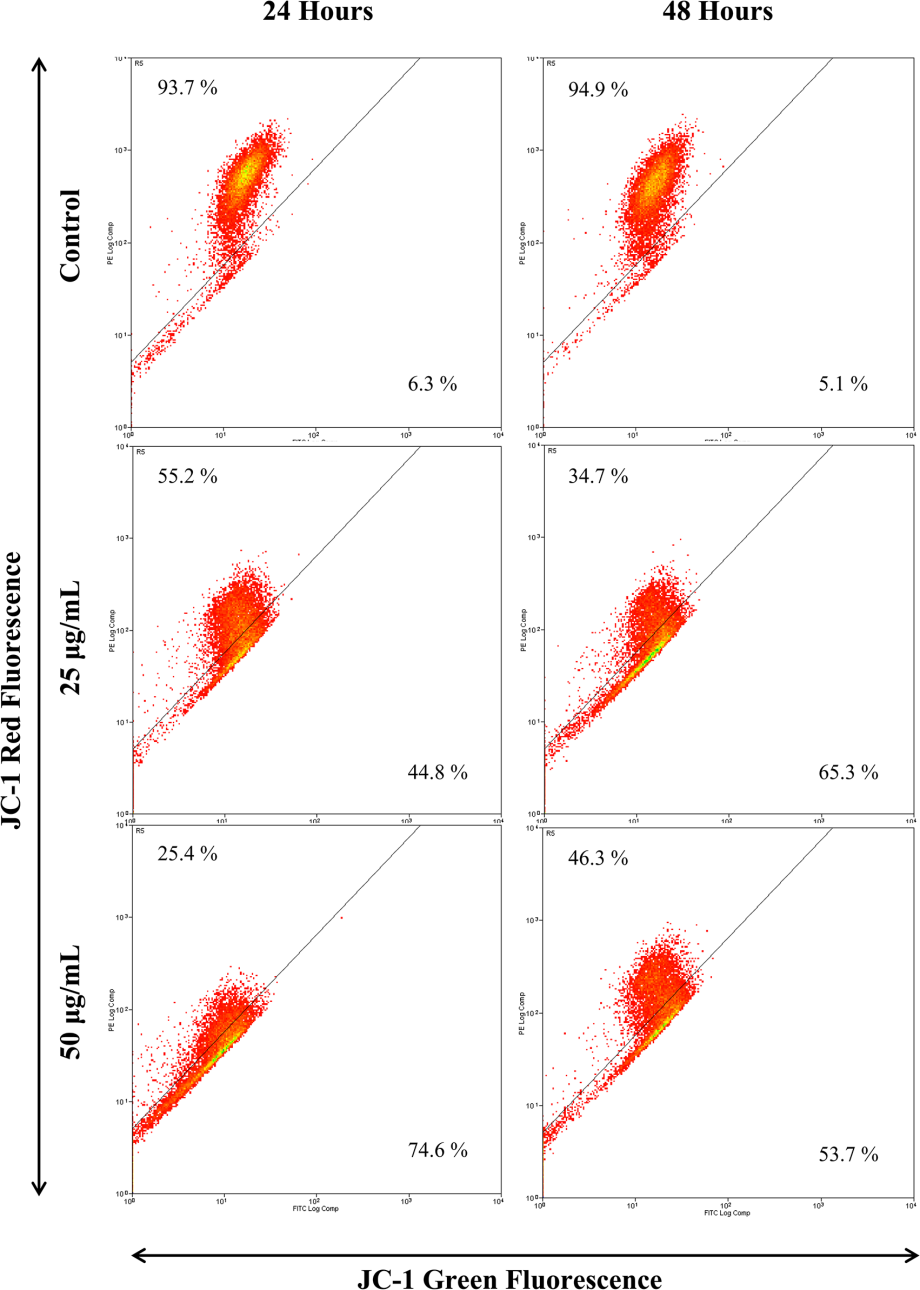
Mitochondria membrane potential of MCF-7 cells treated with EADs as determined by JC-1 fluorescent dye using flow cytometry analysis. The increment of green fluorescence indicates the loss of ΔΨm in the mitochondria of EADs-treated MCF-7 cells. The data are presented as dot plots of JC-1 red fluorescence (Y-axis) against JC-1 green fluorescence (X-axis) of at least three independent tests.

JC-1 fluorescence dye was used to evaluate the permeability of mitochondria membrane in MCF-7 cells treated with EADs. As depicted in Fig 4, following treatment of EADs, the intensity of green fluorescence increased, while the intensity of red fluorescence decreased. At 24 hours, 25 and 50 μg/mL of EADs significantly elevated the green fluorescence to 44.8% and 74.6%, respectively, compared to 6.3% in the untreated control (P<0.05). Similar trend was noted at 48 hours treatment of EADs.

### Effects of EADs on the expression of apoptotic-related genes in MCF-7 cells

**Fig 5:**
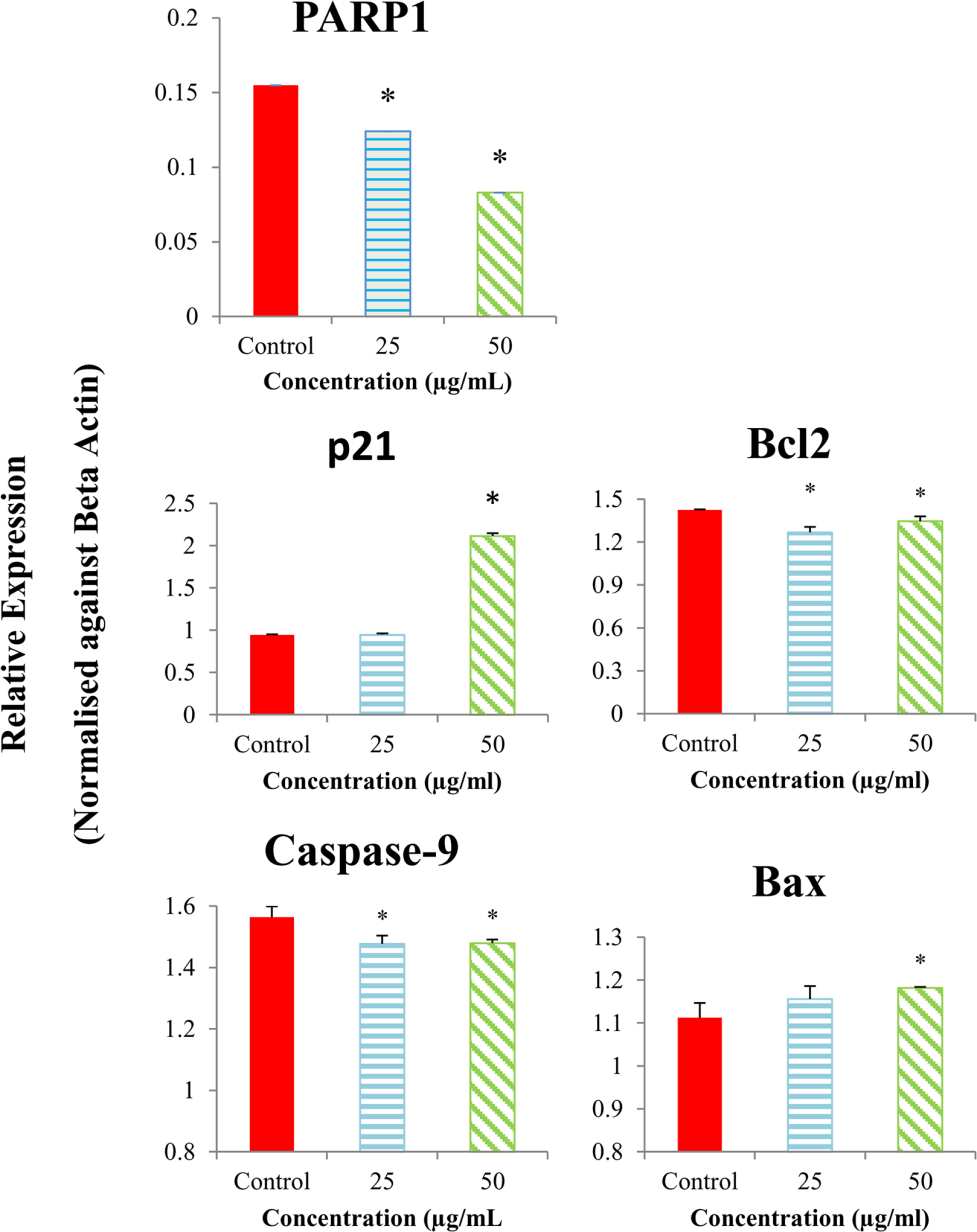
Expression level of the apoptotic-related genes in MCF-7 cells treated with EADs as determined by GeXP analysis. EADs upregulated the expression of *Bax* and *p21* and downregulated the expression of *Bcl-2* and *caspase-9*. The expression of genes was normalized against beta actin and compared to the control. The data are represented as relative expression of genes in bars±SD of at least three replicates from three independent tests. An asterisk * indicates statistically significantly different from the untreated control (P<0.05).

Multiplex GeXP analysis was utilized to measure the effect of EADs on the expression of multiple genes related to apoptotic pathway. As shown in Fig 5, the expression level of *Bax* and *p21* was upregulated, while the exxpression level of *PARP1*, and *Bcl-2* was downregulated following treatment with EADs (P<0.05).

### Effects of EADs on the expression of apoptotic-related proteins in MCF-7 cells

**Fig 6:**
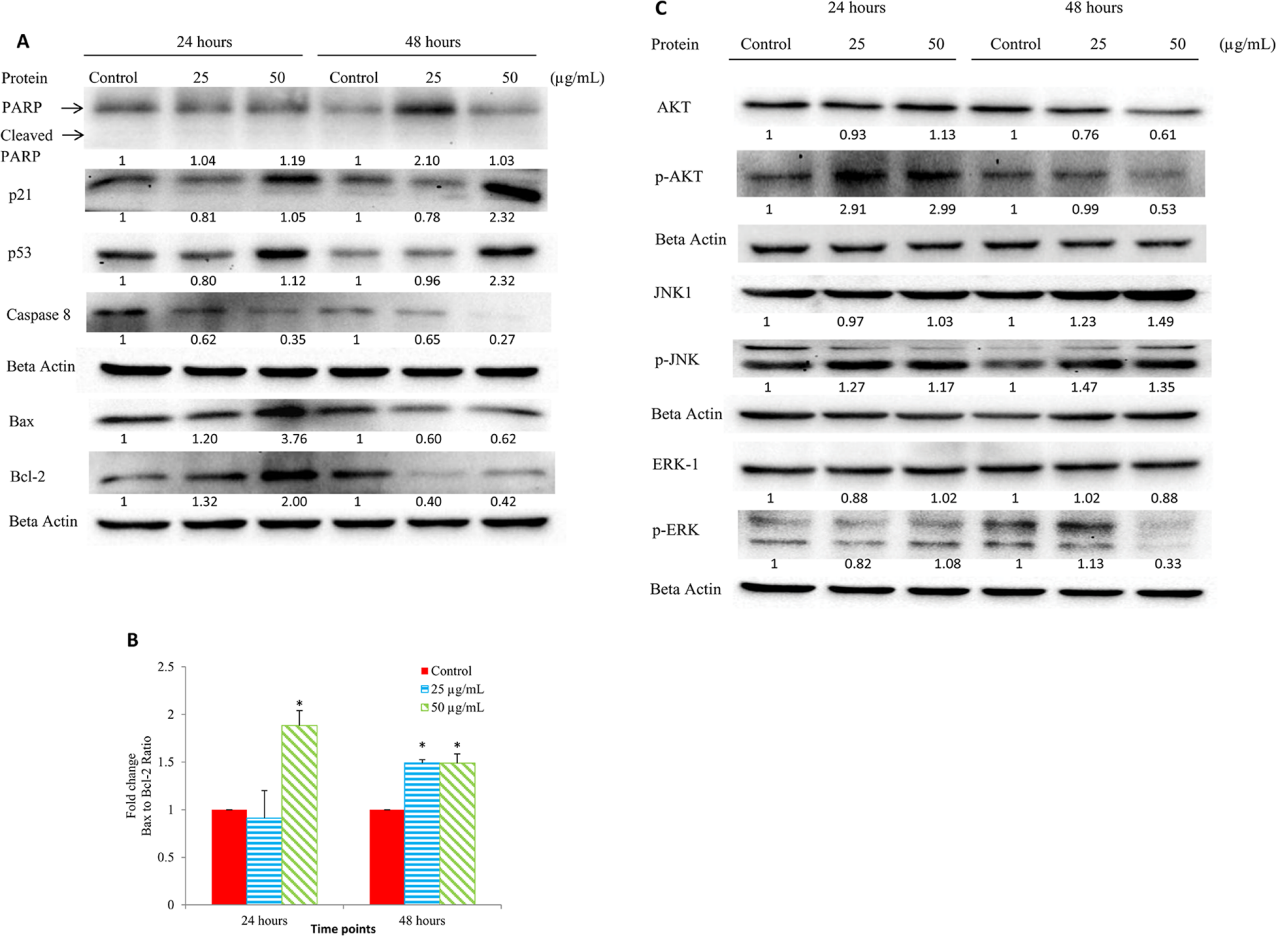
Expression level of the apoptotic-related proteins in MCF-7 cells treated with EADs at different time point as determined by Western blot analysis. (A) Expression of p21, p53, Bax, Bcl-2, PARP and caspase-8 in MCF-7 cells treated with 25 and 50 μg/mL of EADs (B) Fold change of Bax to Bcl-2 ratio at 24 and 48 hours. (C) Expression of AKT-1, phosphor-AKT, JNK-1, phosphor-JNK, ERK-1 and phosphor-ERK1 in MCF-7 cells treated with 25 and 50 μg/mL of EADs. The expression of proteins was normalized against beta actin and compared to the control. The data are represented as mean ± SD of at least three replicates from three independent tests. An asterisk ^a^ indicates statistically significantly different from the untreated control (P<0.05).

Western blot analysis was employed to determine the effect of EADs on the expression of proteins involved in apoptotic pathway. Fig 6A depicts that after treatment with EADs, the expression level of p21 and p53 was upregulated, but caspase-8 was downregulated at 24 and 48 hours. Following treatment with EADs, the expression level of Bax and Bcl-2 was upregulated at 24 hours but downregulated at 48 hours (P<0.05). Fig 6B shows that the ratio of Bax to Bcl-2 in EADs-treated MCF-7 cells was higher compared to the control (P<0.05). The expression of total-Akt was downregulated. Meanwhile, the expression of phosphorylated AKT increased at 24 hours, but declined at 48 hours. The expression level of total-JNK-1 and phosphorylated JNK-1 increased significantly compared to the control at 48 hours (P<0.05) (Fig 6C). On the other hand, significant downregulation in the level of phosphorylated ERK was only observed at 48 hours (P<0.05).

### Compounds of EADs

**Fig 7:**
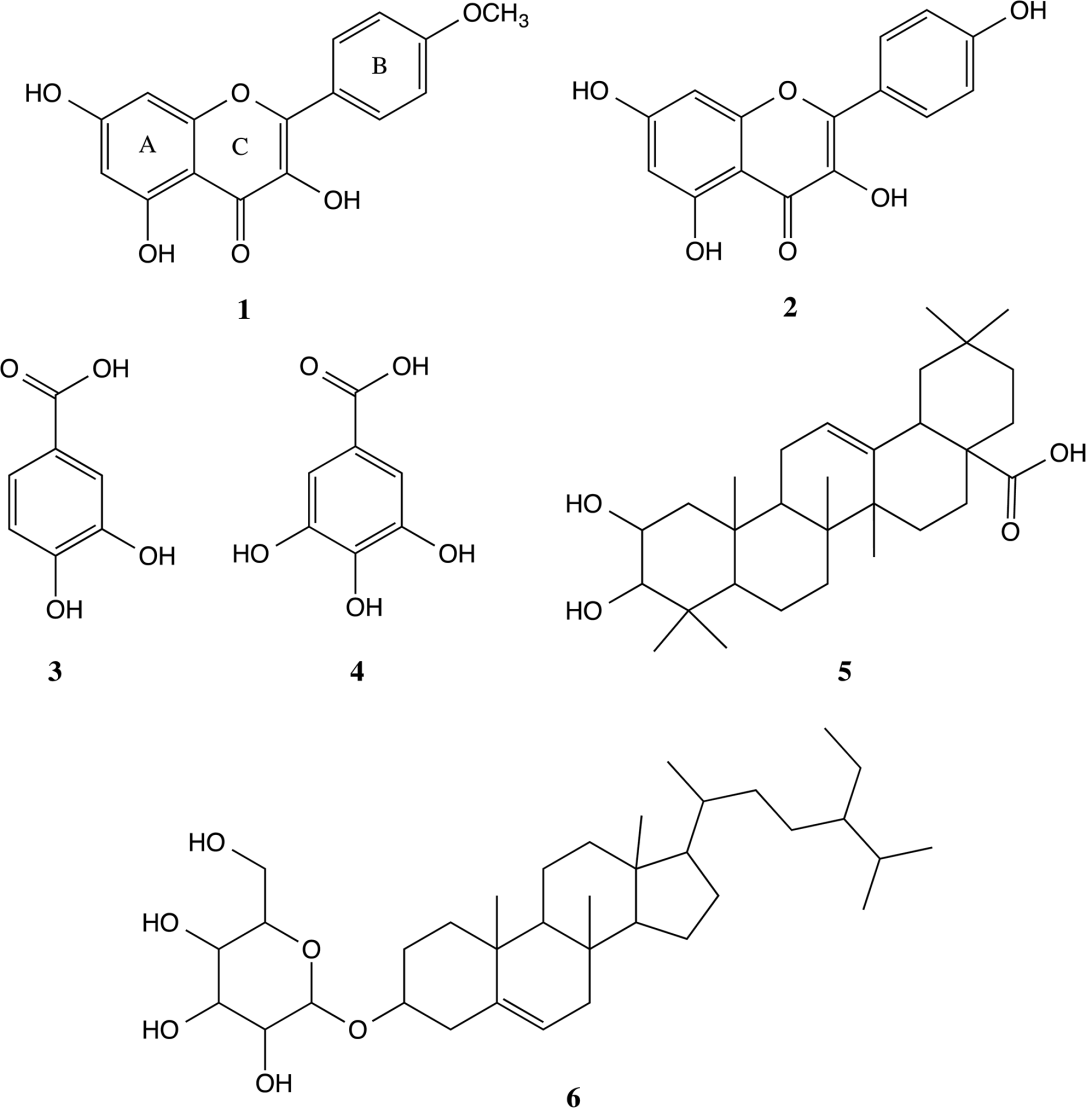
Chemical structure of the compounds isolated from EADs. Structures of the isolated compounds were elucidated using 1^H^ and 13^C^ NMR spectroscopy. The isolated compounds were identified as kaempferide (**1**), kaempferol (**2**), protocatechuic acid (**3**), gallic acid (**4**), 3-epimaslinic acid (**5**) and β-sitosterol-3-O-β-D-glucopyranoside (**6**).

Chemistry profile of EADs was ascertained by isolation of compounds from EADs using column chromatography. The isolation yielded six known compounds including kaempferide (**1**), kaempferol (**2**), protocatechuic acid (**3**), gallic acid (**4**), 3-epimaslinic acid (**5**) and β-sitosterol-3-O-β-D-glucopyranoside (**6**). Structure of compounds was depicted in Fig 7.

#### Compound 1

Kaempferide (3,5,7-trihydroxy-2-(4-methoxyphenyl)chromen-4-one). Yellow powder. ^1^H NMR. (500Mhz, CDCL_3_). δ (ppm): 6.19 (1H, d, *J* = 2.3Hz, H-6), 6.45 (1H, d, *J* = 2.3Hz, H-8), 8.13 (1H, d, *J* = 9.2Hz, H-2’), 7.10 (1H, d, *J* = 9.2Hz, H-3’), 8.13 (1H, d, *J* = 9.2 Hz, H-5’), 7.10 (1H, d, *J* = 9.2Hz, H-6’) 3,86 (1H, s, *J* = 2.3Hz, O-Me). ^13^C NMR (125 MHz, CDCL_3_) δ (ppm): 146.8 (C-2), 136.6 (C-3), 176.5 (C-4), 161.3 (C-5), 98.8 (C-6), 163.6 (C-7), 94.1 (C-8), 156.8 (C-9), 103.6 (C-10), 123.8 (C-1’), 129.9 (C-2’), 114.6 (C-3’), 161.0 (C-4’), 114.6 (C-5’), 129.9 (C-6’), 55.9 (O-Me) [Identical with the reference [22]].

#### Compound 2

Kaempferol (3,5,7-Trihydroxy-2-(4-hydroxyphenyl)-4H-chromen-4-one). Yellow powder. ^1^H NMR. (500Mhz, CD_3_OD). δ (ppm): 6.18 (1H, s, *J* = 2.3Hz, H-6), 6.39 (1H, s, *J* = 2.3Hz, H-8), 8.08 (1H, d, *J* = 9.2Hz, H-2’), 6.90 (1H, d, *J* = 9.2Hz, H-3’), 6.90 (1H, d, *J* = 9.2Hz, H-5’), 8.08 (1H, d, *J* = 9.2Hz, H-6’). ^13^C NMR (125 MHz, CD_3_OD) δ (ppm): 148.1 (C-2), 137.2 (C-3), 177.4 (C-4), 162.6 (C-5), 99.4 (C-6), 165.7 (C-7), 94.6 (C-8), 160.7 (C-10), 123.8 (C-1’), 130.8 (C-2’), 116.4 (C-3’), 158.3 (C-4’), 116.4 (C-5’), 130.8 (C-6’) [Identical with the reference [23]].

#### Compound 3

Protocatechuic acid (3,4-Dihydroxybenzoic acid). Brown crystal. ^1^H NMR. (500Mhz, CD_3_OD). δ (ppm): 7.36 (1H, s, *J* = 2.2 Hz, H-2), 6.71 (1H, d, J = 8 Hz, H-5), 7.34 (1H, dd, J = 9.2 Hz, H-6). ^13^C-NMR (125 MHz, CD_3_OD). δ (ppm): 123.9 (C-1), 117.8 (C-2), 146.1 (C-3), 151.4 (C-4), 115.8 (C-5), 123.9 (C-6), 170.9 (C-7) [Identical with the reference [24, 25]].

#### Compound 4

Gallic acid (3,4,5-Trihydroxybenzoic acid). White crystal. ^1^H NMR. (500Mhz, DMSO-d_6_). δ (ppm): 6.91 (1H, s, H-2), 6.91 (1H, s, H-6). ^13^C-NMR (125 MHz, DMSO-d_6_). δ (ppm): 121.0 (C-1), 109.0 (C-2), 145.9 (C-3), 138.3 (C-4), 145.9 (C-5), 109.0 (C-6), 168.0 (-COOH) [Identical with the reference [26]].

#### Compound 5

3-epimaslinic acid (2α,3α-2,3-dihydroxyolean-12-en-28-oic acid). White crystal. ^1^H NMR. (500Mhz, DMSO-d_6_). δ (ppm): 3.77 (br, dt, J = 2.4; 8.8, H-2), 3.15 (br, d, J = 6.4, H-3), 5.18 (brs, H-12), 1.06 (1H, s, H-23), 0.89 (1H, s, H-24), 0.79 (1H, s, H-25), 0.73 (1H, s, H-26), 1.12 (1H, s, H-27), 0.91 (1H, s, H-29), 0.90 (1H, s, H-30). ^13^C-NMR (125 MHz, DMSO-d_6_). δ (ppm): 41.7 (C-1), 65.2 (C-2), 78.4 (C-3), 38.5 (C-4), 48.1 (C-5), 18.1 (C-6), 32.1 (C-7), 38.6 (C-8), 47.4 (C-9), 38.2 (C-10), 23.6 (C-11), 122.4 (C-12), 144.9 (C-13), 42.2 (C-14), 28.7 (C-15), 26.2(C-16), 43.7 (C-17), 40.2 (C-18), 42.9 (C-19), 29.4 (C-20), 35.0 (C-21), 31.1 (C-22), 28.7 (C-23), 22.4 (C-24), 16.8 (C-25), 17.1 (C-26), 26.9 (C-27), 178.5 (C-28), 32.7 (C-29), 26.3 (C-30) [Identical with the reference [27]].

#### Compound 6

β-sitosterol-3-O-β-D-glucopyranoside. (17-(5-Ethyl-6-methylheptan-2-yl)-10,13-dimethyl-2,3,4,7,8,9,11,12,14,15,16,17-dodecahydro-1H-cyclopenta[a]phenanthren-3-ol). White powder. ^1^H NMR. (500Mhz, DMSO-d_6_). δ (ppm): 3.77 (br, dt, *J* = 2.8; 8.8 Hz, H-3), 2.36 (br, dd, *J* = 2.4; 10.4 Hz, H-4), 5.32 (br, s, H-6), 0.64 (3H, s, H-18), 0.95 (3H, s, H-19), 0.90 (3H, d, J = 7.2, H-21), 0.89 (3H, d, J = 5.6, H-26), 0.79 (3H, d, J = 5.2, H-27), 0.82 (3H, t, J = 5.6, H-29), 4.22 (d, J = 6.0, H-1’), 3.14 (t, J = 6.8, H-2’), 3.02 (t, J = 7.6, H-3’), 2.89 (t, J = 6.4, H-4’), 3.05 (1H, m, H-5’) ^13^C-NMR (125 MHz, DMSO-d_6_). δ (ppm): 37.67 (C-1), 29.21 (C-2), 77.81 (C-3), 37.95 (C-4), 140.46 (C-5), 121.24 (C-6), 33.28 (C-7), 31.37 (C-8), 49.55 (C-9), 36.78 (C-10), 20.53 (C-11), 38.25 (C-12), 41.80 (C-13), 56.21 (C-14), 23.75 (C-15), 27.75 (C-16), 55.37 (C-17), 11.62 (C-18), 18.57 (C-19), 36.16 (C-20), 17.54 (C-21), 35.44 (C-22), 25.78 (C-23), 45.08 (C-24), 28.63 (C-25), 19.05 (C-26), 19.67 (C-27), 21.78 (C-28), 12.07 (C-29) [Identical with the reference [28]].

### Cytotoxicity of the isolated compounds

The cytotoxic effects of the isolated compounds on breast cancer cells were determined using MTT assay. As displayed in Table 2, Compound **4** was the most cytotoxic towards both MCF-7 and MDA-MB-231 cells, with IC_50_ 36 ± 1.7 μg/mL and 35 ± 1.3 μg/mL, respectively, compared to others. Compound **5** was less cytotoxic than Compound **4**, with IC_50_ of 83 ± 2.1 μg/mL, towards MDA-MB-231 cells. Compound **1**, **2**, **3** and **6** were least cytotoxic towards MCF-7 and MDA-MB-231 cells at 72 hours (IC_50_>100 μg/mL).

**Table 2:** Cytotoxicity of the compounds isolated from EADs towards MCF-7 and MDA-MB-231 cells at 72 hours as reflected by IC_50_ value as determined by MTT assay.

Compounds	MCF-7	MDA-MB-231
	IC_50_ (μg/mL)	IC_50_ (μg/mL)
**1**	>100	>100
**2**	>100	>100
**3**	>100	>100
**4**	36 ± 1.7^a^	35 ± 1.3^a^
**5**	>100	83 ± 2.1^a^
**6**	>100	>100

Data are represented as IC_50_ ± SD of three replicates from three independent tests.

^a^ indicates statistically significantly different from untreated control (P<0.05).

## Discussion

There are assortment of signals and cellular metabolic events required to induce apoptosis. One of the cellular events capable of inducing apoptosis is the generation of oxidative stress [29]. Sies [30] defined oxidative stress as “a disturbance in the pro-oxidant to antioxidant balance in favor of the former, leading to potential damage”. In other words, oxidative stress is the outcome of excessive amount of free radicals or reactive species and/or disruption of antioxidant defense. Oxidative stress is tightly related to cancer and is often associated with carcinogenesis, cancer prevention and cancer treatment. Despite the fact that oxidative stress induced by the accumulation of reactive species will enhance tumor growth, it also enhances the sensitivity of cancerous cells to treatment. Evidence showed that reactive species not only function as a regulator of subcellular events but are also able to induce apoptotic cell death [31].

In this study, EADs was found to be less cytotoxic towards MCF-7 cells after pre-treatment with α-tocopherol and ascorbic acid, suggesting that the extract induces oxidative stress as being hypothesized previously [18]. However, the result showed that the inhibition of the cytotoxicity of EADs by both antioxidants decreased at 48 hours. It is deduced that the amount of antioxidants in the cells was depleting over time (Fig 2). The antioxidant activity of α-tocopherol, a lipid-soluble organic compound, is due to the donating ability of its phenolic-hydrogens to lipid free radicals. Thus, α-tocopherol is recognized for the lipid peroxidation inhibition ability [32]. α-tocopherol is capable to cease the chain propagation during lipid oxidation by impairing the oxidizing radicals such as lipid peroxyl radicals, akyl radicals and alkoxyl radicals, which are formed during the oxidation of polyunsaturated fatty acid [33]. Ascorbic acid, which is a water soluble antioxidant, scavenges ROS in aqueous phase by donating electrons via ascorbate peroxidase reaction. Rapid scavenging of ROS will consequently obstruct lipid peroxidation [34, 35]. In addition, ascorbic acid can effectively scavenge hydroxyl radicals and superoxide radicals by oxidation of dehydro-ascorbate [36].

Based on the data from DCFH-DA assay, surprisingly EADS was found to attenuate the ROS formation in MCF-7 cells (Fig 2C). It is suggested that EADs-induced oxidative stress in MCF-7 cells is without the involvement of certain reactive species. This can be explained by DCFH-DA that is not sensitive to other reactive species such as reactive nitrogen species, H_2_O_2_, lipid peroxides, singlet O_2_^-^ and O_2_ [37–39]. Thus, the induction of oxidative stress by EADs could be due to these reactive species. However, this needs further study for confirmation. DCFH-DA is a sensitive free-radical indicator. It is a stable non-polar compound that is readily diffused into cells and oxidized by ROS to form fluorescent 2’, 7’-dichlorofuorescein (DCF), which emits green fluorescence. The intensity of fluorescence is proportional to intracellular ROS level. Reactive species like alkoxyl, peroxyl, carbonate, NO_2_^-^, hydroxyl radical and peroxynitrite, can oxidize DCFH into DCF, which will emit fluorescence signal when excited. Nonetheless, the probe is not sensitive to H_2_O_2_, lipid peroxides, NO^-^, singlet O_2_^-^ and O_2_ [37, 40].

The failure of caspase inhibitor Z-VAD-FMK to suppress EADs-induced apoptosis indicates that the process was caspase-independent (Fig 3). The fact that MCF-7 cells do not express caspase-3 due to the deletion at 47-base pair of this particular gene [41], also indirectly supports the claim that EADs induced caspase-independent apoptosis. Furthermore, decreased activity of *PARP1* and PARP proteolysis that is regulated by caspase activity was also found to be absent in MCF-7 cells treated with EADs, denoting that apoptosis induction by EADs is non-caspase mediated [42]. Results of GeXP and Western blot analysis further confirmed that apoptosis induced by EADs in MCF-7 cells was not caspase-mediated due to decreased activity of caspase-8. Involvement or activation of caspases may be imperative but not exclusive in the determination of apoptosis [43]. Execution of apoptosis can occur in the absence of caspases, with the facilitation of non-caspase proteases like cathepsin, endonuclease and other proteases [44]. Furthermore, exposure of phosphatidylserine and chromatin condensation are not mandatorily followed by activation of caspase effector [45]. Besides, other prominent hallmarks, for instance, cell shrinkage and detachment from substratum can also occur in caspase-independent apoptosis [46].

Previously, EADs has been demonstrated to induce cell cycle arrest in MCF-7 cells [18]. Current data showed that the cell cycle arrest was modulated via activation of p53 and p21 (Fig 5 and Fig 6). The sequence-specific transcriptional activity of p53 is believed to upregulate the expression of p21 in MCF-7 cells. The amplification of p53 and p21 leads to cell cycle arrest and ultimately apoptosis in MCF-7 cells. The tumor suppressor protein, p53, is a crucial regulatory protein in controlling cell growth and cell death. In response to intracellular and extracellular stress, p53 is activated and served as a transcription factor that orchestrates various targets, which in turn modulating multitude of cellular processes such as DNA repair, cell cycle arrest and apoptosis [11, 47]. One of the noteworthy roles of p53 is the ability to induce cell cycle arrest at G_1_, G_2_ or S phase, and enabling DNA repair to take place [48]. p21, a cyclin-dependent kinase inhibitor, will be upregulated by p53 upon cellular stress or DNA damage [49]. Overexpression of p21 can cause cell cycle arrest at G_1_, G_2_ or S phase via interaction with a wide range of cyclin/ CDK complexes [13]. Therefore, activation of p21 mediated by p53 is an important key in altering the cancer cell growth.

Besides the caspase-independent pathway, it is suggested that EADs executes apoptosis via mitochondria-dependent pathway. The elevation of JC-1 green fluorescence indicated the loss of ΔΨm in EADs-treated MCF-7 cells (Fig 4). The dissipation of ΔΨm is attributed to the opening of mitochondrial permeability transition pore (MTP). Hence, it is evidenced that EADs (Fig 6B) has led to the persistent opening of MTP, which resulted in mitochondrial swelling and the rupture of mitochondrial outer membrane, ultimately the release of intermembrane proteins that triggered apoptosis [50]. One of the characteristics of early apoptosis is mitochondrial disruption. Hence, measurement of the ΔΨm can be used as an indicator to determine the involvement of mitochondria in apoptosis induction [51]. ΔΨm is a universal element of cell death and is regarded as ‘point of no return’ in apoptosis induction. Hence, as evidenced by the data in the present study, it is proposed that EADs-induced apoptosis in MCF-7 cells is via mitochondria-dependent pathway by altering the ΔΨm, which consequently leads to a cascade of events driving to apoptosis [52, 53].

**Fig 8:**
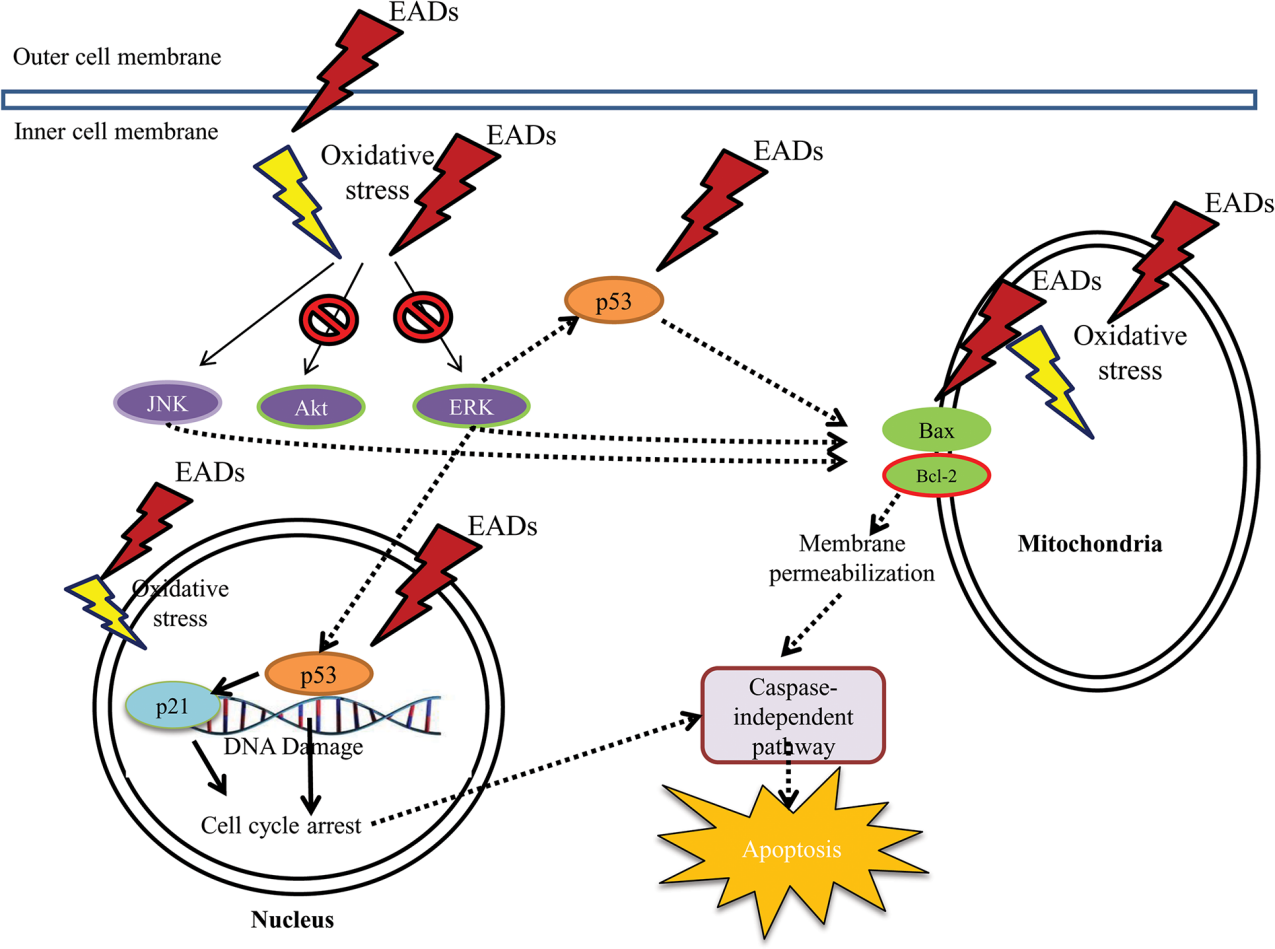
Proposed signalling pathway of EADs-induced apoptosis in MCF-7 cells. It is postulated that EADs induces apoptosis in MCF-7 cells via the production of oxidative stress, p53- and p21-dependent cell cycle arrest, activation of JNK and NF-κB pathways and inactivation of AKT and ERK pathways. Regulation of these pathways eventually leads to the execution of mitochondrial-dependent and caspase-independent apoptosis.

The involvement of the members of MAPK family such as ERK and JNK is also important in the regulation of cancer cells proliferation. It is suggested that modulation of ERK by EADs could indirectly promote the key factors in intrinsic pathway such as the Bcl-2 family members to induce apoptosis via the regulation of Bax and Bcl-2. In addition, the inhibition of ERK was found to trigger apoptosis [54], which is consistent to the finding in this study. Besides, increased activity of phosphorylated JNK in MCF-7 cells by EADs is also believed to upregulate the expression of pro-apoptotic protein, Bax and downregulate the one of anti-apoptotic protein, Bcl-2. Moreover, it is deduced that inhibition of AKT pathway, a cell survival pathway, eventually leads to apoptosis in MCF-7 cells treated with EADs. Apoptotic pathway, either intrinsic or extrinsic apoptotic pathway, is commonly associated with ERK activity [55]. Similar to JNK, ERK activity was reported to modulate the expression of Bcl-2 family members which includes upregulation of Bax, downregulation of Bcl-2 and promotion of the release of cytochrome c. Furthermore, ERK can mediate the phosphorylation and activation of p53 [56]. Additionally, prolonged activation of JNK was reported to be associated with apoptosis [57]. It is reported that triggering of cell proliferation or apoptosis upon JNK activation rely on the stimuli received and the cell-type engaged in the activation [58]. Besides that, PI3K/AKT pathway is an important mediator in regulating cell survival. Study showed that phosphorylation of AKT could abrogate apoptosis in response to the growth factor stimulation [59]. The molecular mechanisms of EADs-induced apoptosis are proposed in Fig 8.

The selectivity of EADs was tested against normal epithelial breast cells, MCF-10A cells. MCF-10A is a non-transformed and immortalized epithelial cell line originated from human fibrocystic mammary tissue. This cell line possesses near diploid karyotype and relies on exogenous growth factors for growing. Its inability to form tumors in nude mice indicates that this cell is non-malignant [60]. An ideal anticancer agent should be able to selectively target cancer cells but render no harm to normal cells. EADs was relatively less cytotoxic to MCF-10A cells (IC_50_ = 60 ± 3.3 μg/ mL) compared to MCF-7 cells (IC_50_ = 39 ± 3.6 μg/ mL) at 72 hours as shown in Table 3. Hence, it indicates that the cytotoxic effect of EADs was more selective towards breast cancer cells compared to the normal breast cells.

**Table 3:** Cytotoxicity of EADs towards the breast cancer and non-breast cancer cell line at different time point as reflected by IC_50_ value as determined using MTT assay.

Cell line		IC_50_ (μg/mL)	
	24 hours	48 hours	72 hours
**MCF-7**	76 ± 2.3^a^	58 ± 0.7 ^a^	39 ± 3.6 ^a^
**MCF-10A**	>100	>100	60 ± 3.3 ^a^

The value of IC_50_ is the average of three replicates from three independent experiments ± SD. The superscript ^a^ indicates statistically significantly different compared to the untreated control group (P<0.05).

Previously, EADs has been reported as a fraction with good antioxidant activities [61], which are believed to be mainly due to the isolated compounds kaempferide (**1**), kaempferol (**2**), protocatechuic acid (**3**) and gallic acid (**4**). Kaempferide (**1**) and kaempferol (**2**), the flavonoids that are commonly found in plants (Fig 6) [62, 63]. Protocatechuic acid (**3**) and gallic acid (**4**) are phenolic acids [64, 65]. Antioxidant effects of plants are commonly attributed to the presence of compounds such as flavonoids and phenolic acids [66]. The antioxidant activity of flavonoids and phenolics is interconnected with their structure, which comprised of phenolic hydrogens that can act as hydrogen-donating radical scavengers [67]. Gallic acid with trihydroxylated structure exhibited high antioxidant activity. Its activity could be explained by the presence of three hydroxyl groups in the compound. This is because the rising degree of hydroxylation will subsequently increase the antioxidant activity [36]. Nonetheless, the structure activity relationship of flavonoids is apparently more sophisticated than phenolic acids due to the relative complexity of the molecules. The antioxidant activity of flavonoids is regarded to the structure and substitutions on the second and third ring of the molecules [68]. As observed in kaempferol (**2**), combination of 3-OH with the double bond between C-2 and C-3 will strengthen the radical scavenging activity of this compound [69].

Apart from that, flavonoids and phenolic acids are ubiquitous in plants, and they have shown promising anticancer and chemopreventive activities through various mechanisms such as cell cycle arrest, induction of apoptosis, suppression of angiogenesis, antioxidant activity and overcoming multidrug resistance [70–72]. Kaempferol has been shown to have remarkable anticancer activities against breast cancer cell line, MCF-7. It induced apoptosis in the cells via activation of mitochondrial pathway by cleavage in PARP, caspase-7 and caspase-9, and accompanied by downregulation of PLK-1 [73]. Kaempferol was also capable to inhibit the expression of estrogen receptor alpha and progesterone receptor in MCF-7 cells [74]. Interruption of the hormone receptors is beneficial in the treatment of estrogen-positive breast cancer metastasis and chemoprevention of breast cancer. Kaempferol also suppressed the vascular endothelial growth factor (VEGF) expression by enhancing the activity of cisplatin in ovarian cancer cells [75]. Gallic acid, also known as 3,4,5-trihydroxylbenzoic acid, has also been demonstrated to induce apoptosis in MCF-7 cells via both extrinsic Fas/FasL and intrinsic mitochondrial pathway [76]. Escalation of p27^Kip1^ and p21^Cip1^ decreased the proliferation of MCF-7 via G2/M phase cell cycle arrest [77]. Another isolated compound, protocatechuic acid, also displayed chemopreventive activity that is associated with its antioxidant activity, by scavenging the generation of reactive radicals in *in vivo* model [78]. Based on the anticancer and chemopreventive properties possess by kaempferol, gallic acid and protocatechuic acid, it is postulated that the cytotoxic effect of EADs towards MCF-7 cells may be due to the presence of these compounds or their synergistic effects in the extract.

## Conclusions

In summary, EADs induced oxidative stress in MCF-7 cells that has led to apoptosis via mitochondria-dependent pathway with the loss of mitochondria membrane permeability. Moreover, EADs induced caspase-independent apoptosis and cell cycle arrest that was facilitated by p21 and p53. Additionally, activation of JNK and inhibition of AKT and ERK pathway play an unequivocal role in EADs-induced apoptosis. This unveils the potential of EADs as an anti-breast cancer agent by modulating multiple pathways involved in the induction of apoptosis.
